# Revising LH cut-off for the diagnosis of central precocious puberty via triptorelin stimulation assay

**DOI:** 10.1007/s12020-024-04055-0

**Published:** 2024-10-09

**Authors:** Paolo Cavarzere, Marco Sandri, Marta Arrigoni, Chiara Guardo, Rossella Gaudino, Franco Antoniazzi

**Affiliations:** 1 Pediatric Division, Department of Pediatrics, University Hospital of Verona, Verona, Italy; 2 Data Methods and Systems Statistical Laboratory, University of Brescia, Brescia, Italy; 3 University of Verona, Verona, Italy; 4 Pediatric Clinic, Department Surgical Sciences, Dentistry, Gynecology and Pediatrics, University of Verona, Verona, Italy; 5 Regional Center for the diagnosis and treatment of children and adolescents rare skeletal disorders. Pediatric Clinic, Department of Surgical Sciences, Dentistry, Gynecology and Pediatrics, University of Verona, Verona, Italy

**Keywords:** Central precocious puberty, LH, Triptorelin Stimulation, GnRH analogues, Cut-off

## Abstract

**Introduction:**

Precocious puberty (PP) in girls is defined by thelarche before age 8. The diagnostic gold standard is an increased LH level following gonadotropin-releasing hormone (GnRH) stimulation. Alternatively, GnRH analogues like triptorelin can be used, though their interpretation varies. Since 2000, we have used a triptorelin-induced LH cut-off of 15 IU/L, 4 h post-stimulus. However, many girls showed LH values below this threshold despite evident pubertal progression.

**Purpose:**

To establish a new LH threshold post-triptorelin stimulation for earlier diagnosis of central precocious puberty (CPP) in girls showing pubertal progression and to evaluate additional parameters for diagnostic accuracy.

**Methods:**

We enrolled 186 girls with thelarche onset between ages 1–8 and a GnRH analogue assay performed between 2015–2019 without signs of axis activation. Within this cohort, 62 patients repeated the triptorelin test due to rapid pubertal progression. The assay involved administering 100 mcg/m² of triptorelin and measuring LH, FSH, and estradiol levels before and four hours post-injection.

**Results:**

Patients with axis activation at the second test had significantly higher post-stimulus LH levels at the first test compared to those below 15 IU/L. They also had higher basal LH levels, elevated LH/FSH ratio, and increased growth velocity. Statistical analysis identified a new post-stimulus LH threshold of 5 IU/L.

**Conclusion:**

We propose a LH value of 5 IU/L after triptorelin administration as a new threshold for early CPP diagnosis. While the LH/FSH ratio and growth velocity are associated with axis activation, they did not significantly enhance diagnostic accuracy when combined with the LH value.

## Introduction

In girls, precocious puberty (PP) is defined as the development of sexual secondary characteristics before the age of 8 years [1, 2].

The early onset of puberty is the result of a precocious activation of the hypothalamic-pituitary-gonadal axis (HPGA), following the physiological pathway. This activation depends on an increasing hypothalamic pulsatile secretion of gonadotropin-releasing hormone (GnRH), and, consequently, of luteinizing hormone (LH) and follicle-stimulating hormone (FSH) from the pituitary gland [3, 4].

The diagnosis of PP in females is determined by evaluating the blood concentration of estradiol, and the levels of LH and FSH, before and after stimulation with luteinizing hormone releasing hormone (LHRH), which appears to be the gold standard test [5]. Nevertheless, the native gonadotropin releasing hormone is too expensive and not available worldwide [6], therefore a feasible alternative involves the employment of GnRH analogues given subcutaneously, followed by the measurement of LH and FSH levels after a standardized time [6–8]. The diagnostic cut-offs depend on the way the test is performed and on the stimulus given; anyhow the current state of the art lacks defined and worldwide approved values to confirm the activation of the HPGA [9, 10]. Recently, some studies have suggested that a basal LH value > 0.3 IU/L indicates pubertal activation. However, in the absence of availability of ultrasensitive assays, basal values often fail to demonstrate pubertal activation, making stimulation tests necessary [5].

In our Center, we routinely use the GnRH analogue stimulation test with triptorelin, with a diagnostic cut-off set for values of LH above 15 IU/L measured four hours after the stimulus. This threshold was established following an unpublished study conducted in our Center in 2002, which compared LH levels obtained after stimulation with LHRH and triptorelin in girls with CPP, performed 7–15 days apart. The threshold value of 15 IU/L was higher than the cut-off of 6 IU/L used with the LHRH test, but allowed all affected patients to be identified. Recently, the 15 IU/L cut-off value has proven to be too high. Many girls exhibit LH levels below this threshold but show clear clinical evidence of incipient puberty and soon develop a full activation of the HPGA. Therefore, it is necessary to reconsider the cut-off established twenty years ago to promptly confirm the diagnosis and implement precocious treatment. A revised threshold would help prevent early menarche leading to a lower final height compared to the prediction based on the familiar target.

This study aims to identify a new LH cut-off value post-triptorelin stimulus in females with CPP. Additionally, it seeks to determine whether other parameters, such as pelvic ultrasonography, increased height velocity and estradiol levels, can aid in diagnosing CPP.

## Materials and methods

### Patients

**Fig. 1:**
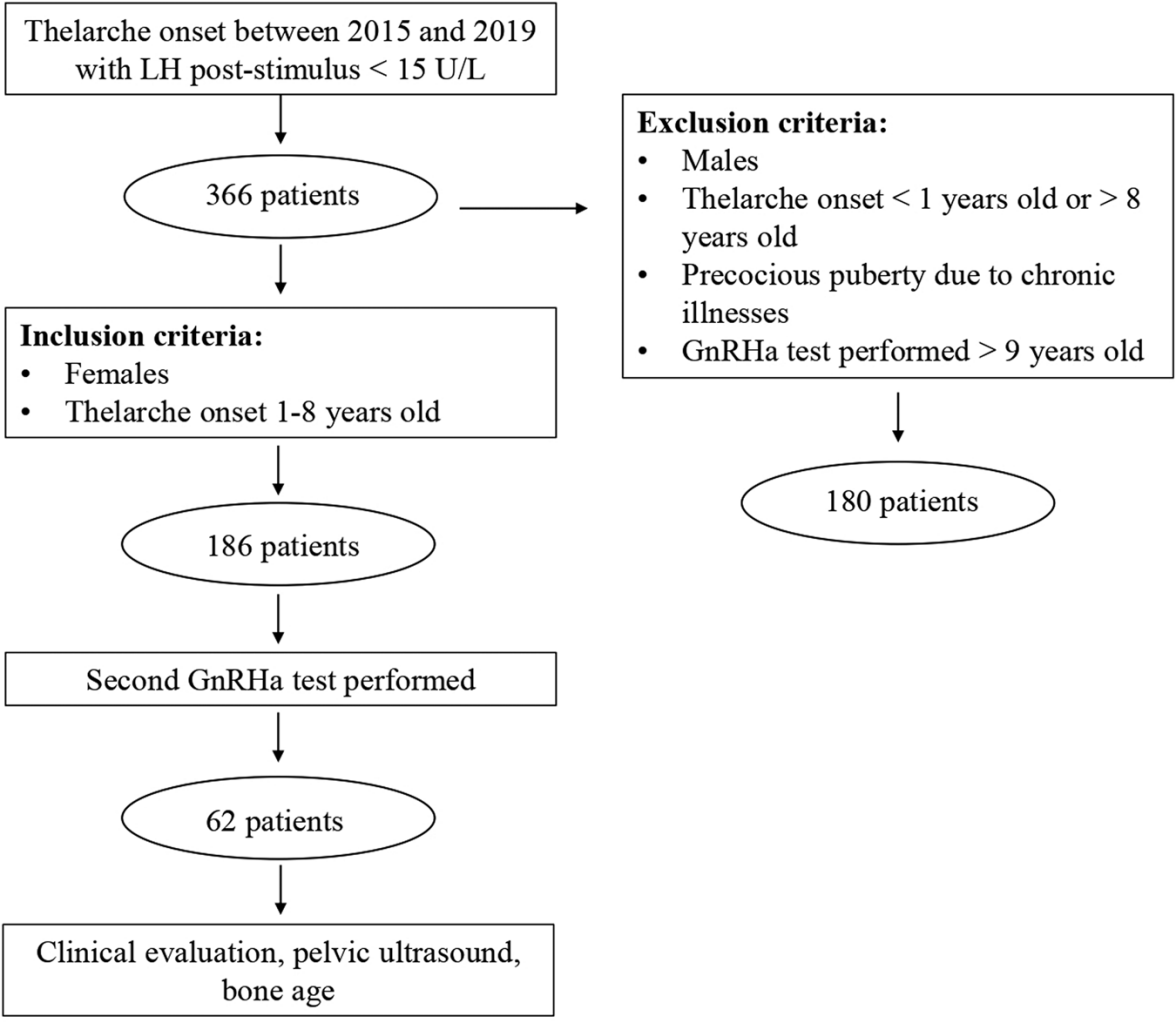
The figure represents the study design

In this retrospective study, we enrolled all children with a clinical suspect of CPP who underwent a GnRH-analogue test with triptorelin, without evidence of HPGA activation, between 2015 and 2019, at the Pediatric Endocrinology Division of the Hospital of Verona, Italy. Among all these patients (n = 366) we excluded male patients, female patients who presented a thelarche before the age of 1 year or after that of 8 years, and those affected by chronic illnesses. Overall, 186 girls met the inclusion criteria. They were re-evaluated periodically every 6 months to assess their clinical development. A stimulation test with triptorelin was repeated when signs of puberty progression were observed, specifically evidenced by an advancement of the Tanner stage and an increase in growth velocity. Figure 1 represents the study design.

For each patient we analysed birth anthropometric measures (weight, length, head circumference and gestational age), data regarding the thelarche and pubarche onset, the hormonal values found during the first GnRH analogous assay, pelvic ultrasound evaluations (longitudinal uterine diameter, antero-posterior diameter and ovarian volume) and bone age. The last parameter was personally evaluated by our team of paediatric endocrinologists using the Greulich and Pyle method [11]. Based on bone age, we could predict the patient’s final height using Bayley and Pinneau tables [12].

During each follow-up appointment, we recorded the patient’s age, height, weight, body mass index (BMI), Tanner pubertal stage and growth velocity. The auxological data were plotted on Cacciari’s growth charts and expressed in units of metric system and in standard deviation scores (SDS) [13]. The pelvic ultrasound was performed transabdominally, considering as indicative of pubertal a longitudinal diameter ≥36 mm and an ovarian volume ≥2 mL (calculated with the formula: $\frac{\pi }{6}$ x longitudinal diameter x anteroposterior diameter x transverse diameter).

The bone age was repeated every year and the pelvic ultrasound every 6 months for those patients that showed signs of pubertal progression during the follow up.

### Assay

The GnRH analogous test was performed with a subcutaneous injection of triptorelin 100 μg/m^2^ (Decapeptyl 0.1 mg, Ipsen, Milan, Italy), with a maximum dose of 100 μg. LH, FSH, and estradiol were measured before the injection and 4 h after the hormonal stimulus.

Serum levels of LH, FSH and estradiol were determined using a solid phase chemiluminescence immunoassay (Immulite 2000, Siemens Healthcare Diagnostic, USA), with analytic sensitivities of 0.05 IU/L for LH, 0.1 IU/L for FSH and 15 pg/mL for estradiol. The intra-assay and inter-assay variability coefficients were 3.60% and 6.70% for LH, 2.90% and 4.10% for FSH and 11.70% and 13% for estradiol, respectively.

### Statistical analysis

Continuous variables were reported as mean ± standard deviation (SD), while categorical variables were expressed as counts and percentages.

To evaluate differences in continuous variables between independent groups, the t-test was utilised, while Fisher’s exact test was employed to compare categorical variables.

The ability of the patients’ clinical, auxological, and biochemical characteristics to predict the activation of the HPGA in the second assay was evaluated using logistic regression analysis. This approach enables the calculation of odds ratios (ORs) and the area under the receiver operating characteristic curves (AUC-ROC), thereby providing an assessment of the predictive strength of each parameter examined.

All analyses were conducted using STATA version 16 (StataCorp. 2019. College Station, TX). A p-value of less than 0.05 was considered to indicate statistical significance.

## Results

The median age at the onset of pubarche and thelarche was 6.87 ± 0.92 years and 7.21 ± 0.82 years, respectively (*p* < 0.001).

In our study population, 12.8% were prematurely born girls; however, there was no statistically significant difference in the age of thelarche and pubarche onset between these patients and those born at term.

Obese patients constituted 9.7% of the study group, whereas overweight patients made up 33.3%. Although no statistically significant difference was found in the age of thelarche onset between normal-weight and overweight patients, as well as between overweight and obese patients, a significant difference was observed between normal-weight and obese children (7.28 ± 0.79 years vs. 6.83 ± 0.93 years, *p* = 0.039).

All auxological data, related to birth, and to the first and second evaluations, are reported in Table 1. At the first evaluation, the bone age was found to be 1.12 ± 0.96 years greater than the patients’ chronological age. Consequently, the average adult height prediction was reduced to 159.36 ± 7.53 cm, approximately 5.04 ± 7.26 cm lower than the family genetic target (*p* < 0.001). The mean uterine length, measured via pelvic ultrasound during the first visit, was 33.63 ± 5.82 mm. Additionally, the other parameters measured included antero-posterior uterine thickness (8.71 ± 2.65 mm), right ovarian volume (1.26 ± 0.58 mL), and left ovarian volume (1.19 ± 0.53 mL).

**Table 1:** Patient’s auxological data (related to birth, first and second evaluations)

	DATA AT BIRTH (*n* = 186)	FIRST EVALUATION (*n* = 186)	SECOND EVALUATION (*n* = 62)
Age (years)		7.79 ± 0.83	8.44 ± 0.82
Height (cm)	49.30 ± 2.79	131.45 ± 7.50	136.49 ± 7.11
SDS height	0.28 ± 1.24	0.84 ± 1.07	1.04 ± 1.08
Weight (kg)	3.07 ± 0.65	31.89 ± 6.72	35.05 ± 8.46
SDS weight	−0.05 ± 1.03	0.59 ± 0.95	0.64 ± 1.00
BMI (kg/m^2^)	-	18.33 ± 2.80	18.63 ± 3.01
SDS BMI	-	0.40 ± 0.97	0.36 ± 0.94

Of the enrolled girls, 66% continued with the follow-up, while the remaining 34% discontinued the recommended clinical course. Following the second evaluation, complete regression of thelarche was observed in 11% of patients, and significant progression in another 11%. Among them, 6.7% were confirmed as obese, and 26.9% were overweight. Bone age assessments conducted at the second evaluation for 14.5% of patients revealed an average age of 10.35 ± 1.10 years, an increase of 1.51 ± 0.81 years compared to their chronological age. For 35% of patients, pelvic ultrasounds were repeated, showing the longitudinal diameter of the uterus to be 38 ± 7.24 mm, a significant increase from the first evaluation (*p* < 0.001). The antero-posterior uterine thickness measured 12.28 ± 5.85 mm, with the right ovarian volume at 1.32 ± 0.70 mL and the left ovarian volume at 1.35 ± 0.67 mL.

Sixty-two repeated the GnRH analogue test approximately 1.04 ± 0.74 years later; in 20 of these patients, the LH value measured 4 hours after the GnRH stimulus exceeded 15 IU/L, prompting the initiation of replacement treatment. Among them, 55% had LH values greater than 5 IU/L in the initial test.

Table 2 summarises the auxological, instrumental, and hormonal parameters of patients demonstrating HPGA activation versus those whose post-stimulus LH did not exceed the cut-off. Notably, patients showing HPGA activation at the second assay had significantly higher post-stimulus LH values in the initial triptorelin test (*p* = 0.003). Additionally, girls with axis activation at the second test exhibited significantly elevated basal LH (*p* < 0.001), basal estradiol (*p* = 0.023), post-stimulus estradiol (*p* < 0.001), and LH-to-FSH ratio (*p* < 0.001) compared to their prepubertal counterparts. Moreover, the uterine longitudinal diameter was significantly greater in the former group than in the latter (*p* = 0.032), indicating a higher growth velocity between the two evaluations (*p* = 0.034).

**Table 2:** Auxological data, instrumental evaluations and hormonal results at the first and second GnRH analogue tests collected for patients with a 4-hour post-stimulus LH (from the second test) below and above 15 IU/L

	4 h POST-STIMULUS LH ≥ 15 IU/L (SECOND TEST)		4 h POST-STIMULUS LH < 15 IU/L (SECOND TEST)	
	*First* *GnRHa test*	*Second GnRHa test*	*First* *GnRHa test*	*Second GnRHa test*
Height (cm)	130.15 ± 8.52	137.99 ± 7.68	131.07 ± 6.95	135.75 ± 6.79
SDS height	0.51 ± 1.43	0.75 ± 1.30	1.11 ± 1.00	1.18 ± 0.94
Weight (kg)	30.14 ± 7.98	34.90 ± 9.21	31.76 ± 6.98	35.13 ± 8.18
SDS weight	0.15 ± 1.39	0.28 ± 1.13	0.77 ± 0.92	0.81 ± 0.89
Growth velocity (cm/year)	-	7.10 ± 1.65	-	5.88 ± 2.25
SDS growth velocity	-	1.71 ± 1.96	-	0.29 ± 2.89
BMI (kg/m^2^)	17.69 ± 4.17	18.11 ± 3.44	18.37 ± 2.86	18.89 ± 2.93
SDS BMI	0.03 ± 1.28	0.01 ± 1.04	0.48 ± 0.90	0.52 ± 0.86
Bone age (years)	8.96 ± 0.99	11.25 ± 1.23	8.54 ± 1.32	9.91 ± 1.17
Δ(bone age – chronological age) (years)	1.12 ± 0.96	1.60 ± 0.91	1.25 ± 1.10	1.41 ± 0.85
Uterine longitudinal length (mm)	34.95 ± 6.64	41.68 ± 8.58	34.72 ± 5.78	35.91 ± 6.52
Uterine anteroposterior length(mm)	8.38 ± 2.70	13.33 ± 4.82	8.82 ± 3.23	11.90 ± 8.82
Right ovarian volume (mL)	1.26 ± 0.73	1.63 ± 0.81	1.28 ± 0.59	1.48 ± 0.79
Basal LH (IU/L)	0.25 ± 0.26	2.80 ± 2.36	0.23 ± 0.21	0.43 ± 0.66
Post-stimulus LH (IU/L)	5.53 ± 3.48	39.50 ± 26.46	3.30 ± 2.21	5.31 ± 3.96
Basal FSH (IU/L)	2.31 ± 1.35	4.77 ± 2.15	1.71 ± 1.25	2.79 ± 4.54
Post-stimulus FSH (IU/L)	22.72 ± 7.06	23.19 ± 6.95	21.38 ± 10.22	19.94 ± 9.84
LH/FSH	0.25 ± 0.17	1.71 ± 0.87	0.16 ± 0.10	0.30 ± 0.31
Basal estradiol (pmol/L)	94.63 ± 60.61	120.25 ± 80.34	91.63 ± 67.79	74.22 ± 68.60
Post-stimulus estradiol (pmol/L)	111.96 ± 69.52	216.94 ± 121.48	93.20 ± 58.27	82.27 ± 57.27

The ability of the above-mentioned parameters, to predict whether the HPGA activation was assessed using logistic regression analysis. According to Table 3, uterine length, bone age progression, and estradiol levels, were not significantly associated with the diagnosis of CPP. In contrast, growth velocity (AUC-ROC = 0.63, *p* = 0.016), LH/FSH ratio (AUC-ROC = 0.67, *p* = 0.019), and post-stimulus LH (AUC-ROC = 0.70, *p* = 0.004) were identified as significant predictors for PP.

**Table 3:** Assessing the discriminative power of auxological variables and instrumental evaluations (evaluated at the first GnRH analogue test) for subjects with LH values above and below the 15 IU/L threshold; univariate odds ratios (with 95% CI and p-value) and area under the ROC curve for each parameter

	ODD RATIO (95% CI)	AUC-ROC CURVE (95% CI)	*P*
Age at puberty onset (years)	1.10 (0.55–2.22)	0.57 (0.40–0.73)	0.8
Height SDS	0.63 (0.36–1.10)	0.64 (0.47–0.81)	0.1
Weight SDS	0.59 (0.33–1.05)	0.65 (0.49–0.82)	0.074
BMI SDS	0.64 (0.34–1.21)	0.64 (0.47–0.80)	0.2
Bone age (years)	1.34 (0.87–2.08)	0.61 (0.46–0.77)	0.2
Δ(bone age – chronological age) (years)	0.89 (0.51–1.53)	0.55 (0.39–0.72)	0.7
Uterine length (cm)	1.01 (0.92–1.11)	0.51 (0.35–0.67)	0.9
Basal LH (IU/L)	1.24 (0.09–17.3)	0.45 (0.30–0.61)	0.9
**Post-stimulus LH (IU/L)**	1.33 (1.09–1.61)	0.70 (0.55–0.84)	**0.004**
Basal FSH (IU/L)	1.42 (0.91–2.22)	0.63 (0.47–0.79)	0.1
Post-stimulus FSH (IU/L)	1.02 (0.96–1.07)	0.56 (0.41–0.71)	0.6
**LH/FSH**	1.90 (1.11–3.25)	0.67 (0.52–0.82)	**0.019**
Basal estradiol (pmol/L)	1.00 (0.99–1.01)	0.54 (0.38–0.70)	0.9
Post-stimulus estradiol (pmol/L)	1.00 (0.99–1.01)	0.58 (0.41–0.74)	0.3
**Growth velocity (cm)**	1.40 (1.06–1.83)	0.63 (0.48–0.78)	**0.016**

*AUC-ROC* area under the ROC curve, *CI* confidence interval, *P* *p*-value from the Wald test assessing the significance of the odds ratio.

Bold values: *p*<0.05

**Fig. 2:**
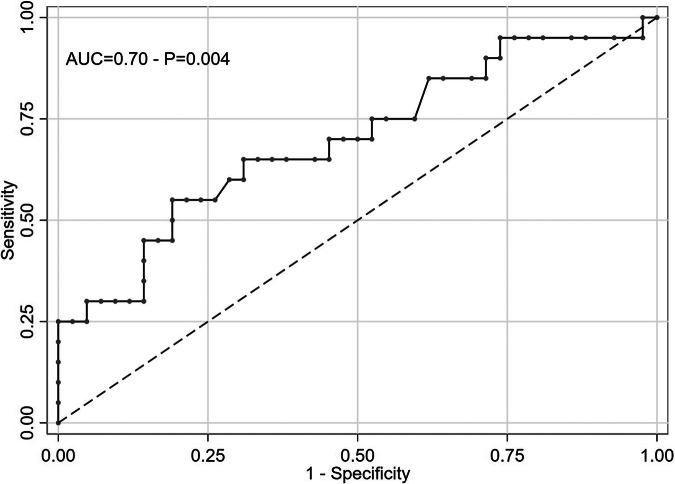
Correlation amongst LH levels after the first and second test

Based on the above statistical analysis, we propose to modify the cut-off for the 4 h post-stimulus LH to a value of 5 IU/L, achieving a sensitivity of 55% and a specificity of 79%, as shown in Fig. 2.

## Discussion

This study identifies a value of 5 IU/L as a possible new LH threshold after GnRH analogous assay with triptorelin, in the diagnosis of CPP in girls with thelarche onset before 8 years old. Only a few studies have analyzed the LH level after a triptorelin test for the diagnosis of PP and we believe that our data may be useful for a better definition of this test. As a matter of fact, the proposed cut-off value of 5 IU/L corresponds to the value suggested by the Italian Society of Pediatric Endocrinology and Diabetology guidelines on the basis of the 2009 consensus statement on the use of gonadotropin-releasing hormone analogues in children [14], confirming the validity of our result.

It is well-known that the gold standard for the diagnosis of central PP is represented by LH levels measurements after a stimulation test. However, different cut-offs to set the diagnosis have been proposed according to the test used, the time of assessment and the population analyzed [14].

So far, few data aiming to a standardized interpretation of the subcutaneous triptorelin assay have been presented in literature, and a great variability has emerged among assays. A retrospective study proposed an LH post stimulus cut-off of 6 IU/L collecting serial blood samples at baseline and at 30, 60, 90, 120 minutes after the triptorelin stimulus [15]. On the contrary, a prospective case-control study tested 46 girls with premature thelarche using both intravenous GnRH test and subcutaneous triptorelin assay. Gonadotropin levels were measured at 0, 3 and 24 h after the stimulus, proposing a cut-off of 7 IU/L (with immunofluorometric assay - IFMA) or 8 IU/L (with electrochemiluminescence immunoassay - ECLIA) to differentiate premature thelarche from central PP [7]. This study also highlighted the usefulness of the 24 h estradiol dosage in identifying pubertal evolution in borderline cases. A third prospective study has recently proposed a cut-off of LH post-stimulus measured at 180 minutes of 3.4 IU/L [16]. Finally, a Korean study suggested a peak of stimulated LH level ≥4.5 IU/L at 120 min after triptorelin injection as indicative of CPP [17].

Although all these studies have adequately demonstrated the diagnostic value of triptorelin, there is no consensus regarding the appropriate blood sampling intervals and the time for LH peak determination. This is due to different causes, and first of all, to pharmacokinetic differences in the LH response after intravenous gonadorelin administration, compared to the triptorelin test following subcutaneous injection. Consequently, the time required for the gonadotropin to reach its peak concentration depends on the type and on the modality of administration of the stimulus used. Secondarily, the drug levels following the subcutaneous injections may be influenced by the injection volume, the tissue distribution of proteolytic enzymes, and the regional variation in blood flow. Moreover, triptorelin acetate is known to have a longer half-life and a stronger affinity with the GnRH receptor compared to gonadorelin. As a final point, triptorelin manages to increase the plasma concentration of GnRH for a longer period compared to gonadorelin, and therefore the LH level should be measured over a longer period of time in girls with suspected CPP. Our cut-off results are similar to those proposed by other studies but our method involves a 4-hour control, hence an exact comparison is not possible, given that previous studies proposed cut-off values measured 2 or 3 h after the stimulus. Our Centre was the first to use the triptorelin test in humans, using 0.1 mg/m^2^ of triptorelin subcutaneously and analysing blood samples for LH, FSH, and estradiol 4 h after the injection, based on the data of a previous study involving five healthy adult volunteers, which demonstrated that gonadotropins show its peaks 4 h after triptorelin administration [18]. An LH value of 15 IU/L, dosed 4 h after the subcutaneous triptorelin stimulus, has been routinely used as cut-off for over two decades to diagnose CPP, without any reported missed diagnoses. The value of 15 IU/L derived from a comparison of post-stimulus LH levels after LHRH and triptorelin stimulus in a cohort of girls (n = 108) with CPP compared with other girls with isolated premature thelarche without activation of the HPGA (unpublished data). Lately, it has been noticed that some patients with clinically progressive CPP showed an LH value below the diagnostic threshold and needed a second assay after a few months to confirm the diagnostic hypothesis. The method used for the assay in our Centre was a chemiluminescence immunoassay, which has remained unchanged for the last twenty years; the variation in the progression of the PP was therefore independent from the method used. Hence the decision to review the cut-off for this test. In fact, these girls underwent a watchful wait approach through clinical follow-up, which in some cases led to a delay in the beginning of the replacement therapy.

The decision whether to treat or not a girl for CPP must be taken individually after assessing both the age of onset of secondary sexual characters and the evidence of pubertal progression during follow-up. The degree of bone age advancement and uterine dimensions on pelvic ultrasound may also be helpful in the diagnostic process and in the decision to start the treatment. The most notable long-term complications of an untreated CPP are represented by growth impairment and by a final height below the predicted familiar target [19], as well as by several psychological consequences of premature menarche, which, despite being often difficult to analyse, can persist over time, modifying self-perception, even in adulthood. Since puberty and adolescence are particularly vulnerable periods for the emotional chances they bring on, another reason for a rapid treatment of CPP lies in a psychosocial discomfort, which might lead to precocious sexual activity and to the risk of early pregnancies [10, 19]. In fact, a prompt treatment allows to preserve the growth potential and to reach the familiar height target avoiding psychological issues.

Our data show that other useful parameters to assess the diagnosis of CPP, such as the uterine longitudinal diameter, the bone age progression and the estradiol levels, provide useful indicators from a clinical viewpoint but are not decisive for establishing a diagnosis. Actually, our data confirm that pelvic ultrasound alone is not indicative of CPP and should be combined with clinical and laboratory tests to maximise its diagnostic value in the diagnosis of CPP [20]. In fact, although uterine and ovarian measurements are significantly higher in girls with CPP, there is a significant overlap of normal prepubertal and early pubertal values [21]. Moreover, none of the girls enrolled in this study shows evidence of HPGA activation at the first assay, consequently the role of pelvic ultrasound for them is even more limited. Finally, we did not measure the 24-hour estradiol value because the double sampling appeared to be too expensive, long and above all not easy for our patients.

Interestingly, our population presents an advancement, although not significant, of bone age in comparison with the chronological age. This data may be of difficult interpretation. It may be related to an activation of the hypothalamic-pituitary-gonadal axis, or to be a consequence of being overweight. In fact, it is well-known that obesity could lead to a bone age progression due to the aromatization of androgens into estrogens in the adipose tissue [22, 23].

In our cohort we enrolled some overweight and obese girls, and we noticed that these patients developed thelarche earlier compared with the normal weight girls, with an average advancement of 0.45 years (7.26 ± 0.80 years in normal weight girls vs 6.81 ± 0.92 in obese girls, *p* < 0.05). These data confirm what previous literature has already shown: obese patients tend to begin the pubertal developmental process earlier than normal weight girls do. Unfortunately, data concerning the relationship between central PP and patients’ nutritional status are lacking and it is difficult to establish whether a higher BMI influences the earlier onset of puberty or, on the contrary, whether CPP could be partially responsible for the excessive weight gain [10].

Beyond post-stimulus LH values, other variables resulted significantly different amongst girls with a precocious activation of the HPGA, such as LH-to-FSH ratio and growth velocity. For what concerns LH-to-FSH ratio, in literature values between 0.6 and 1 have been proposed as cut-offs to distinguish a progressive CPP from a non-evolutive one [24]. Our study demonstrates that this ratio could be useful to support the diagnosis, even though the main biochemical diagnostic value remains the LH levels. Remarkably, even growth velocity represents a significant parameter to detect progressive CPP. Yet since this variable must be evaluated at least in a 6-months period, it becomes useful only during the auxological follow-up and not at the first evaluation of the patient. Therefore, growth velocity might be something to take into consideration during clinical follow-up without necessarily repeating a stimulus test.

This study employs a retrospective design and includes patients who did not exhibit HPGA activation in the initial assay. This distinction might account for some discrepancies between our findings and those of other studies, as we hypothesise that patients with clear pubertal activation would demonstrate significant bone age advancement and larger uterine longitudinal diameters. Despite these limitations, our objective is not to compare patients with CPP and isolated premature thelarche but rather to identify the most appropriate LH value that could obviate the need for repeated GnRH analogous tests during follow-up in girls previously exhibiting only partial HPGA activation.

Even though further studies are needed to better define a universal cut-off for LH post-stimulus with GnRH analogues, the present article allows to identify a potential new threshold for LH levels measured 4 h after the subcutaneous triptorelin administration of 5 IU/L in a cohort of Italian girls with thelarche onset before 8 years old. Other parameters that appeared to be statistically significant and that might support the activation of the HPGA are the LH/FSH ratio and the growth velocity; yet, when considered together with the LH value, they did not significantly enhance the diagnostic capability. The other variables we hypothesised as useful in supporting the diagnosis of PP, such as the uterine longitudinal diameter, the bone age advancement and estradiol levels, are not statistically significant to set the diagnosis.

## Data Availability

All the results generated are provided in this article, raw data analysed during this study can be enquired to the corresponding author.
